# The PeptideAtlas of a widely cultivated fish *Labeo rohita:* A resource for the Aquaculture Community

**DOI:** 10.1038/s41597-022-01259-9

**Published:** 2022-04-13

**Authors:** Mehar Un Nissa, Panga Jaipal Reddy, Nevil Pinto, Zhi Sun, Biplab Ghosh, Robert L. Moritz, Mukunda Goswami, Sanjeeva Srivastava

**Affiliations:** 1 Department of Biosciences and Bioengineering, Indian Institute of Technology Bombay, Powai, Mumbai, 400076 India; 2 Institute for Systems Biology, Seattle, WA 98109 USA; 3 Central Institute of Fisheries Education, Indian Council of Agricultural Research, Versova, Mumbai, Maharashtra 400061 India; 4 Regional Centre for Biotechnology, Faridabad, 121001 India

**Keywords:** Protein-protein interaction networks, Agriculture

## Abstract

*Labeo rohita* (Rohu) is one of the most important fish species produced in world aquaculture. Integrative omics research provides a strong platform to understand the basic biology and translate this knowledge into sustainable solutions in tackling disease outbreak, increasing productivity and ensuring food security. Mass spectrometry-based proteomics has provided insights to understand the biology in a new direction. Very little proteomics work has been done on ‘Rohu’ limiting such resources for the aquaculture community. Here, we utilised an extensive mass spectrometry based proteomic profiling data of 17 histologically normal tissues, plasma and embryo of Rohu to develop an open source PeptideAtlas. The current build of “Rohu PeptideAtlas” has mass-spectrometric evidence for 6015 high confidence canonical proteins at 1% false discovery rate, 2.9 million PSMs and ~150 thousand peptides. This is the first open-source proteomics repository for an aquaculture species. The ‘Rohu PeptideAtlas’ would promote basic and applied aquaculture research to address the most critical challenge of ensuring nutritional security for a growing population.

## Abstract (summary)

**Table:** 

Measurement(s)	Proteins and Peptides
Technology Type(s)	Mass Spectrometry
Sample Characteristic - Organism	*Labeo rohita*
Sample Characteristic - Location	India

## Background & Summary

The average annual increase in global consumption of fish has outpaced population growth. Of the global animal protein consumption, 20% is met by fish suggesting the importance of fish in global food security and nutrition. India ranks second in global aquaculture production and Indian major carps (IMCs) contribute to more than 75% of its aquaculture economy^1^. *Labeo rohita* (Rohu) is an IMC and among the top eleven finfish species produced in world aquaculture^1^. With the emergence of genomic information for Rohu, this species has entered the post-genomic era such as transcriptomics, proteomics and metabolomics research to address key issues like safety, quality and health in aquaculture.

Proteomic approaches have been applied in diverse areas to investigate developmental biology, physiology, disease mechanisms, impact of stress inducers^2^ and effects of dietary supplements on overall physiology of fish^3,4^. Application of proteomics studies in zebrafish and *Xiphophorus sp*. has revealed the role of phosphorylated Ezrin in gastrulation^5^ and peroxiredoxins in human melanoma^6^. Proteomics can identify and explore sensitive and specific markers for assessing the quality of fish or fishery related products^7^. The effect of pesticide mixtures and temperature have also been explored in goldfish (*Carassius auratus*)^8^. All these findings suggest the importance of proteomic characterization of fish in addressing basic biological to ecological, environmental and food related issues.

Mass spectrometry (MS) based proteomic approaches are progressively used to disentangle complex biological questions, often associated with other omics disciplines (e.g., genomics, transcriptomics, metabolomics)^9,10^. Proteome reference maps for many organisms such as human and zebrafish have been generated using high resolution mass spectrometry^11–13^. A recent publication of Rohu genome reported a prediction of 26,400 protein coding genes^14^. However, proteomics studies in Rohu are rare with most studies focusing on only a particular tissue in isolation^15,16^.

Data repositories like PeptideAtlas^17^, PRIDE^18^ and Global Proteome Machine Database^19^ enable successful planning of MS-based experiments for biomedical research. The PeptideAtlas project mainly provides a large collection and precise analysis of available MS-based proteomics data. With the exception of the model organism, Zebrafish, no other aquaculture species is well represented so far in any of the publicly available proteomics databases. Towards this goal, an extensive proteomic profiling of 17 histologically normal tissues in Rohu, embryo and plasma was performed using high-resolution high-mass accuracy mass spectrometry. Here, we provided mass spectrometric evidence of more than 150 thousand peptides corresponding to 6015 high confidence canonical proteins with 1% FDR. This dataset has been utilised to develop the PeptideAtlas repository for Rohu. To our knowledge, this is the first such extensive open-source peptide dataset for Rohu.

This work could be considered as a basis for proteomic research on specific genes related to fish health by studying various aspects like improvement in fertility, muscle quality and molecular alterations during stress conditions^20^. The PeptideAtlas interface is user friendly and very useful in designing targeted proteomic experiments by evaluating the candidate peptides or transitions suited for targeted proteomics based diagnostic assays for fish disease, safety and quality. Using this dataset, spectral libraries can be generated for designing and validating the targeted proteomics data. We believe this extensive proteomic sequence information would complement the genomic information allowing basic and applied research to move faster in fisheries and aquaculture sectors.

## Methods

### Fish collection and acclimatisation

Three-month old healthy *L. rohita* fingerlings of around 10 ± 2 g weight, were collected from Powarkheda Regional Centre of ICAR-CIFE, Madhya Pradesh, India. Laboratory conditions used for fingerling acclimatisation included aeration 24 h, daylight 12 h, 10% daily water exchange, water temperature 28–30 °C and feeding twice by 2% of body weight. Following an acclimatisation of seven days, five healthy fishes were placed in an aquarium under starving conditions for one day followed by euthanization for sample collection. Nineteen different types of samples were collected as shown in Table 1 which includes one whole embryonic tissue sample, blood plasma and 17 tissues. Fifteen of the tissues were collected from fingerlings whereas plasma and gonadal tissues from adult fishes. Blood plasma was collected from female fish and embryos were sampled after four days of fertilisation. Collected samples were stored at −80 °C till further use.

**Table 1:** Tissue types and sampling details.

S. no.	Sample	Collection stage
**1**	**AB (Air bladder)**	Fingerling (10 ± 2 g)
**2**	**Brain**	
**3**	**Eye**	
**4**	**Fin**	
**5**	**GB (Gall bladder)**	
**6**	**Gill**	
**7**	**Gut**	
**8**	**Heart**	
**9**	**Kidney**	
**10**	**Liver**	
**11**	**Muscle**	
**12**	**Scale**	
**13**	**Skin**	
**14**	**SC (Spinal cord)**	
**15**	**Spleen**	
**16**	**FG (Female gonad)**	Adult female (1000 ± 100 g)
**17**	**Plasma**	
**18**	**MG (Male gonad)**	Adult male (1000 ± 100 g)
**19**	**Embryo**	4-day post fertilisation

### Protein extraction for in-depth proteomic profiling

For extraction of proteins, organ wise samples collected from individual fish were pooled and taken forward. For lysing the tissue, urea buffer containing 8 M Urea, 50 mM Tris-HCl, 1 mM MgCl_2_ and 75 mM NaCl was used. For fifteen of the tissues including spleen, spinal cord, skin, scales, muscle, male gonad, liver, kidney, heart, gut, gill, female gonad, eye, brain and air bladder, pH shift solubilisation method^20^ was used for protein extraction. For these tissues, proteins were extracted using urea buffer in three different pH i.e., pH 2.5, 8 and 13. To around 75–100 mg of tissue sample, 300 µl of lysis buffer was added followed by sonication for 2-3 times (Vibra-Cell™ Ultrasonic Liquid Processors, VCX 130 (Sonics). The sample was bead beated using Zirconium/Silica beads (Cat. No. 11079110z) for 90 s. It was followed by centrifugation at 8000 rpm at 4 °C for 15 min to get a clear supernatant containing proteins. For the embryo sample, whole embryos were processed using Trizol method^21^ of protein extraction. Plasma sample were directly (without any depletion) taken for downstream analysis.

### Protein quantification and quality check on SDS-PAGE

Protein quantification was performed by Bradford protein assay, using Bovine Serum Albumin (BSA) as a standard. Accordingly, absorbance was taken at 595 nm and standard curve was plotted using BSA dilutions and concentration for all the unknown samples was determined. In order to check the quality of the protein extract, 1-dimensional SDS-PAGE was performed for which 15 ug protein was loaded for each sample onto a mini-vertical gel (Bio-Rad Mini PROTEAN® 3 Cell, Bio-Rad Laboratories), in accordance to Laemmli protocol^22^. As the extracted protein was present in urea containing buffer, no heating step was performed before SDS-PAGE to avoid the risk of Carbamylation. Gel electrophoresis was performed for 1-2 hours followed by staining in Coomassie blue R350 solution in methanol and acetic acid. Gel was destained to visualise the protein bands (Supplementary Fig. S1a).

### Fractionation, in-gel digestion and peptide preparation

**Fig. 1:**
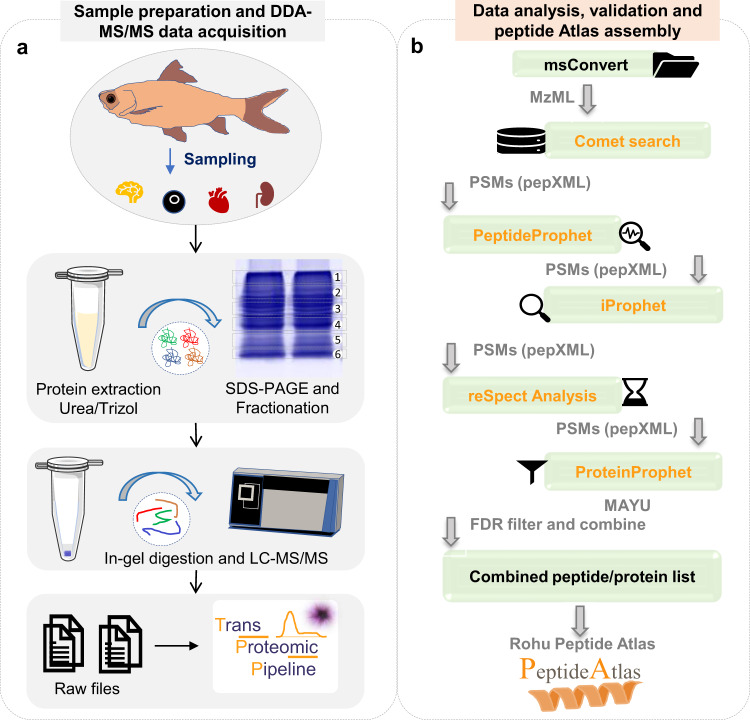
An overview of experimental design and analysis workflow. (**a**) Fishes were dissected to collect the tissue/samples followed by protein extraction and SDS-PAGE. Gel slices were excised and processed for in-gel based tryptic digestion followed by Liquid chromatography tandem mass spectrometry (LC-MS/MS) and analysis in Trans proteomic pipeline (TPP), (**b**) Raw data obtained from DDA-MS were processed along the pipeline for building PeptideAtlas. Raw files were first converted to mzml followed by comet search and analysis pipeline including peptide prophet, reSpect, iPROphet, protein prophet and final filtering and validation to compile the atlas.

For in-gel digestion, 30 µg protein from each sample was run on SDS-PAGE as above. Each sample was run in duplicate and at least six slices per lane were excised (Fig. 1a). For plasma sample, 11 gel fractions were processed for in-gel digestion. The electrophoresis was performed for only 30–40 minutes i.e., ~1 cm in the resolving gel. Before performing the digestion of protein, stain was removed followed by protein reduction and alkylation. For removing stain from the gel pieces an alternate treatment with buffer salt ammonium bicarbonate (NH_4_HCO_3_) and organic solvent Acetonitrile (ACN) solution was performed. Proteins were reduced using Dithiothreitol (DTT) and alkylated using Iodoacetamide (IAA). For protein digestion, trypsin was used in ~1:30 enzyme to protein (w/w) ratio. Peptides were extracted from the gel pieces after 16–18 hours of digestion using an increasing gradient of ACN solution. Peptides were desalted using C18 Empore™ SPE Disks matrix (Merck). Peptide quantification was done using Scopes method^23^ and one µg of peptide was subjected to mass spectrometric analysis.

### Data-dependant Acquisition by Liquid Chromatography Tandem Mass spectrometry (LC-MS/MS)

An Easy-nLC nano-flow liquid chromatography 1200 system was used for the separation of peptides following in-gel digestion (Fig. 1a). With a flow rate of 5 µl/min, one µg desalted peptides were loaded to pre-analytical column (Thermo Scientific, PN 164564-CMD, Trap column nanoViper C18, 5 µm, 100 Å, Acclaim PepMap 100- 100 µm x 2 cm). The peptides were run over a gradient of 120 min in solvent B which was a solution of 80% ACN with 0.1% Formic acid (FA). The flow rate was kept as 300 nl/min for resolving peptides on the analytical column (Thermo Scientific, PN ES903, C18- 75 μm × 50 cm, 2 μm particle, PepMap RSLC, 100 Å pore size). Mass spectrometric data was acquired using Orbitrap mass analyser in DDA mode in a full scan range of 375–1700 *m/z* at a mass resolution of 60,000. For dynamic exclusion, the mass tolerance was set as ± 10 for 40 s and for MS2 precursors, the isolation mass window was set to 1.2 Da. High energy Collision Dissociation (HCD) method was used for MS/MS fragmentation. For MS1 and MS2, AGC target was set to be 400000 and 10000, respectively. A lock mass of 445.12003 m/z was used for positive internal calibration.

The mass spectrometric data used in this study for developing PeptideAtlas of *Labeo rohita* has been utilised for tissue wise profiling of post-translational modifications (PTMs) and comparative protein expression analysis as reported in our recent study^24^.

### Protein identification, TPP analysis and PeptideAtlas assembly

The raw mass spectrometry data (.raw) generated from the Orbitrap Fusion mass spectrometer was converted to .mzML files using MSconvert 3.0.5533 tool^25^. The converted mzML files were searched using Comet (2019.01 rev.1)^26^ tool against *L. rohita* NCBI protein database. This database consisted of protein sequences generated by translation of coding sequences (CDS) through gene predictions after whole genome sequencing of *Labeo rohita* (Bio project: PRJNA437789). The database had locus tag IDs (prefix Rohu_) and EMBL/Bank/GenBank/DDBJ CSS IDs (prefix RXN). UniProt database for this species (ProteomeID- UP000290572) consists of a UniProt protein identifier for each CD. The NCBI database had 32687 entries and the UniProt database which was downloaded on 16^th^ August, 2019, has 32379 entries and is the subset of the NCBI database. For initial comet search, NCBI database was used whereas all downstream steps including protein identification and PeptideAtlas assembly were performed using combined database of NCBI and UniProt. We utilized the combined database so that the proteins which are not yet included in the UniProt database, can also be covered in PeptideAtlas build.

To the protein database, an equal number of decoy and contaminant sequences were added. Decoy sequences were generated using “randomize sequences and interleave entries” decoy algorithm whereas the contaminant sequences were taken from common Repository of Adventitious Proteins, cRAP, database (http://www.thegpm.org/crap/). The parameters used for the data analysis in Trans-Proteomic Pipeline (TPP) suite include peptide mass tolerance 20 ppm, fragment ions bin tolerance 0.05 *m/z* and monoisotopic mass offset 0.0 *m/z*, two allowed missed cleavages, fully tryptic and semi-tryptic peptides, oxidation of tryptophan and methionine (+15.994915 Da) as variable modifications and carbamidomethylation of cysteine (+57.021464 Da) as static modification. Protein identification was performed using TPP V 5.2.0 Flammagenitus^27^. To score for peptide spectral match (PSM), integrated tools of PeptideProphet and iProphet were used for individual files and the score unique peptides in combined PeptideProphet files. Finally, ProteinProphet tool was used for protein identification based on iProphet input and true identifications were selected at less than 1% FDR^28–30^. The whole workflow is represented in Fig. 1b.

The chimeric spectra were accessed by reanalysing the iProphet files using reSpect algorithm^31^. In brief, reSpect search was performed on iProphet files by increasing the precursor mass tolerance to 3.0 Da. TPP analysis was performed as mentioned earlier and the process of reSpect and TPP analysis was repeated once. A minimum iProphet probability ≥ 0.0 was used for the reSpect search. PeptideAtlas processing pipeline was used to build PeptideAtlas by combining the iProphet results from regular TPP and reSpect search results. The spectrum was filtered at variable probability to get constant peptide spectrum match (PSM) FDR of 0.0008% for each experiment. The statistically significant results were organized in the “Rohu PeptideAtlas”, which is built and maintained by ISB at the given link. http://www.peptideatlas.org/builds/rohu/.

#### Ortholog analysis for the identified proteome

Ortholog analysis for the total canonical proteins was performed in EGGNOG-mapper genome-wide functional annotation tool^32^ (http://eggnog-mapper.embl.de/). Firstly, the FASTA sequences were acquired from UniProt^33^ of all the protein IDs and taken as input list (Supplementary Table S1). During this analysis, taxonomic scope was selected as Actinopterygii, orthology restrictions selected as ‘transfer annotation from any ortholog’, seed ortholog detection criteria were set to be 0.001.

### Acquisition of selected reaction monitoring (SRM) data for targeted verification

The targeted proteomic data was acquired using a Thermo TSQ Altis Triple Quadrupole Mass Spectrometer linked to a Thermo Vanquish HPLC system. The data was acquired using an SRM/ MRM (Selected/ Multiple reaction monitoring) acquisition mode. A Hypersil GOLD analytical column (Thermo Fisher Scientific, 100 × 2 mm, C18) was used for the reverse phase separation of peptides. Samples were run at a flow rate of 450 µl/ min. One µg of desalted peptide sample was subjected to the column and run for 10 minutes. The liquid chromatography system used, consisted of 0.1% formic acid (FA) in milliQ water as solvent A and 80% Acetonitrile (ACN) and 0.1% FA as solvent B. Throughout the run, the column temperature was set to be 45 ^ο^C and cycle time was kept as 2 s. The Skyline daily software^34^ (version 20.2.1) was utilised for analysing the data.

## Data Records

### Data record 1

Mass spectrometry data obtained after DDA-MS experiments includes raw files (.raw) for 19 different sample types of fish (Supplementary File S1). This mass spectrometry data along with the protein databases (.fasta) has been deposited to the ProteomeXchange Consortium via the PRIDE partner repository and can be accessed through the identifier PXD026377 using the link https://www.ebi.ac.uk/pride/archive/projects/PXD026377^35^. The comet search parameter file and MAYU statistical report (.xlsx) is provided in Supplementary File S2 and S3 respectively. Peptides identified are enlisted in Supplementary Table S2. The details of the proteins and peptides identified along with various interactive data and visualizations are available at PeptideAtlas and can be accessed using the given link https://db.systemsbiology.net/sbeams/cgi/PeptideAtlas/buildDetails?atlas_build_id=500^36^.

### Data record 2

The targeted mass spectrometry data includes spectral library files (.blib), the target peptide list selected based on PeptideAtlas data (.xls), instrument raw files (.raw) and the result imported skyline documents (.sky, .view, .skyd, .skyl). The targeted proteomics data including all skyline documents, raw files and spectral library have been deposited to Panorama web server^37^. Also, the target peptides and transition lists are given in supplementary Tables S3 to S5.

### Data record 3

**Fig. 2:**
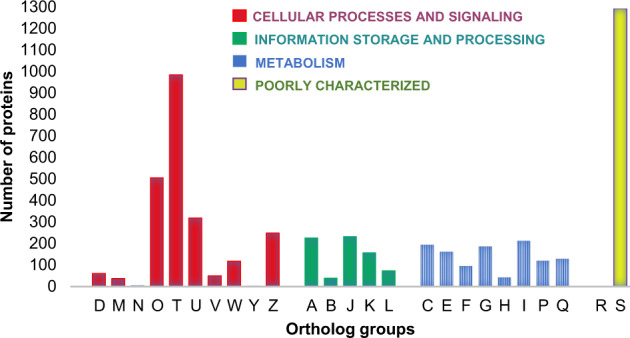
An overview of phylogenetically annotated orthologs for the canonical proteins. The distribution of identified proteins mapped against each ortholog group is presented here (ortholog details in the Table 2).

In the EggNOG database^32^ based ortholog analysis, the canonical proteins were mapped against orthologs corresponding to wide range of cellular processes and metabolic functions. Around 97% of the mapped orthologs belong to *Actinopterygii*, the class of ray finned fishes and majority of them were linked to signal transduction mechanism. This information is represented in Fig. 2/ Table 2 and Supplementary Table S1).

**Table 2:** Distribution of identified canonical proteins across various orthologs*.

Groups	Description	No. of proteins
**CELLULAR PROCESSES AND SIGNALING**		
**D**	Cell cycle control, cell division, chromosome partitioning	62
**M**	Cell wall/membrane/envelope biogenesis	38
**N**	Cell motility	5
**O**	Post-translational modification, protein turnover, and chaperones	505
**T**	Signal transduction mechanisms	982
**U**	Intracellular trafficking, secretion, and vesicular transport	318
**V**	Defense mechanisms	50
**W**	Extracellular structures	119
**Y**	Nuclear structure	4
**Z**	Cytoskeleton	249
**INFORMATION STORAGE AND PROCESSING**		
**A**	RNA processing and modification	226
**B**	Chromatin structure and dynamics	40
**J**	Translation, ribosomal structure and biogenesis	232
**K**	Transcription	158
**L**	Replication, recombination and repair	74
**METABOLISM**		
**C**	Energy production and conversion	195
**E**	Amino acid transport and metabolism	163
**F**	Nucleotide transport and metabolism	96
**G**	Carbohydrate transport and metabolism	187
**H**	Coenzyme transport and metabolism	43
**I**	Lipid transport and metabolism	213
**P**	Inorganic ion transport and metabolism	120
**Q**	Secondary metabolites biosynthesis, transport, and catabolism	129
**POORLY CHARACTERIZED**		
**R**	General function prediction only	0
**S**	Function unknown	1289

^*^This data is in continuation of data represented in Fig. 2.

## Technical Validation

### Building and validation of an extensive PeptideAtlas for *Labeo rohita*

Targeted proteomics is an emerging approach for acquiring proteome wide qualitative and quantitative information in a targeted manner. Generally, the targeted proteomics involves a hypothesis driven experiment which starts from a list of precise protein/peptide targets to be monitored. PeptideAtlas is a compendium of peptides that can serve as an important resource for designing a targeted experiment or validating the protein/peptide target related to a shotgun experiment. To generate the PeptideAtlas resource for Rohu, the DDA-MS dataset was analysed using a combined non-redundant Uniprot database and NCBI database of *Labeo rohita* (details in the Methods). To make the data more reliable, accurate and to avoid the identification of false positives, we used MAYU^38^ tool both at the protein and peptide level. Mayu is a software used to determine false discovery rates (FDRs) for protein identification (protFDR), peptide identification (pepFDR) and peptide-spectrum match (mFDR). All experiments were thresholded at a probability that yields an iProphet model-based PSM-FDR of 0.0008%. The exact probability varies from experiment to experiment depending on how well the modeling can separate correct from incorrect. However, this probability threshold is typically greater than 0.99. For each experiment, the spectra were filtered at variable probability to get constant PSM level FDR of 0.0008%. Throughout the procedure, decoy identifications were retained and then used to compute final decoy-based FDRs. The model-based PSM-FDR was adjusted if the final decoy-based protein FDR is higher than 1%. For protein identification, based on iProphet input, true identifications were selected at less than 1% FDR.

**Fig. 3:**
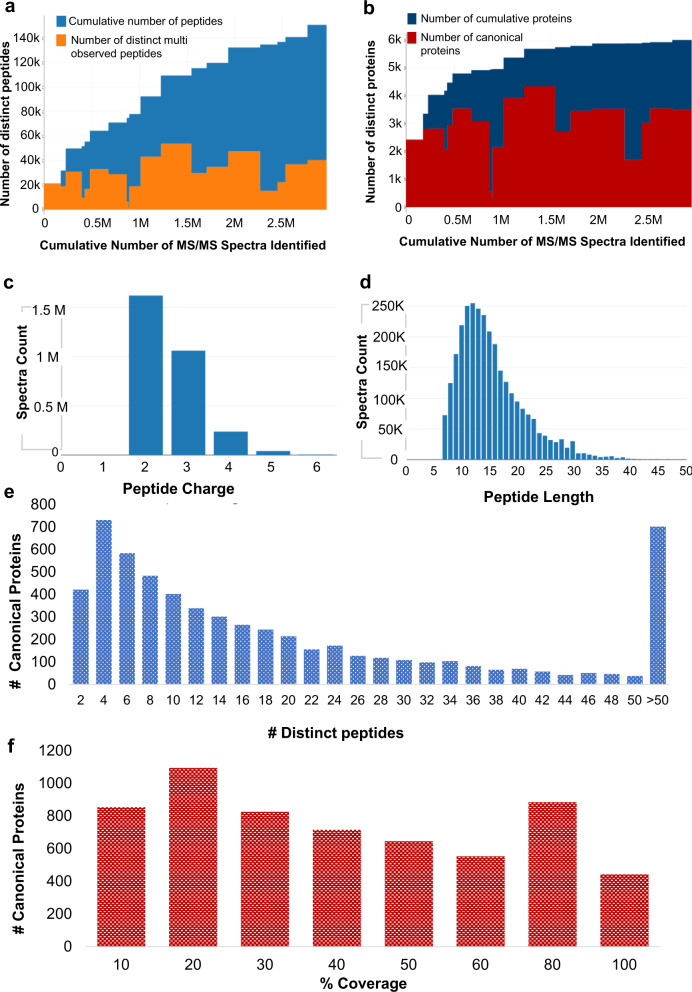
An overview of *Labeo rohita* PeptideAtlas build. (**a,b**), Plots showing cumulative number of peptides and canonical proteins respectively contributed by each experiment. Height of the blue/navy blue bar represents cumulative number of peptides/proteins, height of the orange/red bar represents number of peptides/proteins identified in each experiment and width of the bar (x-axis) represents the number of spectra identified (PSMs) for each experiment, (**c**) Distribution of peptide spectral matches against the peptide charge, (**d**) Graph showing the spectral count for the peptides of different lengths and (**e**). Bar plot representing the number of unique peptides (distinct peptides) per canonical protein where the x-axis shows the bins for number of unique peptides and y-axis show the number of respective canonical proteins, (**f**) Distribuition of canonical proteins based on percentage sequence coverage [Fig. 3a–e are taken from ‘Experiment Contribution Plots’ section of first page of *Labeo rohita* PeptideAtlas].

This resulted in the identification of 6015 high confident canonical proteins along with 667 indistinguishable representative proteins, 671 marginally distinguished proteins, 768 representative proteins and 1165 other proteins. The overall summary for Rohu PeptideAtlas is shown in Table 3. Briefly, the current build contains more than 2.96 million identified peptide MS/MS spectra with additional information for a selection of PSMs at FDR level less than or equal to 0.0008% (i.e., 150781 distinct peptides at 0.18% peptide level FDR) (Fig. 3a). This peptide information corresponds to all the identified proteins at less than 1% protein level FDR. All tissues except muscle, fin, scale and plasma have contributed ~15,000-20,000 peptides and ~2000–3000 canonical proteins each to the build (Fig. 3b). Majority of the identified peptides were doubly or triply charged with a length of 10–20 amino acids and most of the identified peptides were without any missed cleavage (Fig. 3c,d, Supplementary Fig. S1b). Each canonical protein has at least 2 unique peptides and ~93% of them had at least ≥3 unique peptides (Fig. 3d, Table S2). As far as the sequence coverage is concerned, observed peptides for ~54% of the canonical proteins spanned >30% of the protein sequence whereas 22% of canonical proteins had >60% coverage (Fig. 3e, Table S2). PeptideAtlas is a user-friendly portal for researchers who can access protein and peptide related information. The Rohu PeptideAtlas hence provides a platform for obtaining detailed information of all identified proteins and peptides that can be helpful for discovery experiments as well as designing targeted assays for *L. rohita*.

**Table 3:** Organ wise numerical summary for the data in *Labeo rohita* PeptideAtlas.

Dataset	Experiment Tag	MS Runs	Spectra Searched	Distinct Peptides	Unique Peptides	Cumulative Peptides	Distinct Canonical Proteins	Unique Canonical Proteins	Unique All Proteins	Cumulative Canonical Proteins
PrePX245	Air bladder	18	1073739	21148	1754	21147	2428	10	66	2428
PrePX245	Embryo	6	342968	18801	1063	31710	2813	14	108	3360
PrePX245	Female gonad	24	1299928	30777	6364	49703	2828	53	194	4038
PrePX245	Fin	6	421953	9329	274	51602	2074	2	44	4188
PrePX245	Gallbladder	9	489743	16708	690	55308	2953	7	77	4478
PrePX245	Gill	18	1999739	32801	2706	64160	3552	17	222	4808
PrePX245	Gut	18	1005859	28610	2781	70910	3105	12	147	4929
PrePX245	Female plasma	8	430788	6497	1743	74623	562	0	19	4954
PrePX245	Scales	15	710427	1225	123	74888	323	0	7	4956
PrePX245	Skin	13	776257	18838	944	77876	2164	1	17	4970
PrePX245	Spinal cord	18	1093689	43051	4862	92280	3920	28	169	5380
PrePX245	Brain	18	1113115	53736	13205	109378	4343	190	868	5698
PrePX245	Eye	18	797200	29665	4328	115355	2727	35	116	5757
PrePX245	Kidney	18	1131662	34743	2527	119636	3467	15	107	5803
PrePX245	Liver	18	1203630	47487	10469	132274	3544	40	282	5887
PrePX245	Muscle	21	1034502	15167	2288	134681	1697	5	37	5894
PrePX245	Spleen	16	817942	22059	1999	136880	3036	11	145	5916
PrePX245	Heart	18	1042352	36798	3751	140791	3554	16	160	5936
PrePX245	Male gonad	18	847818	40259	9990	150781	3501	79	277	6015

### Protein and peptide search in *Labeo rohita* PeptideAtlas

**Fig. 4:**
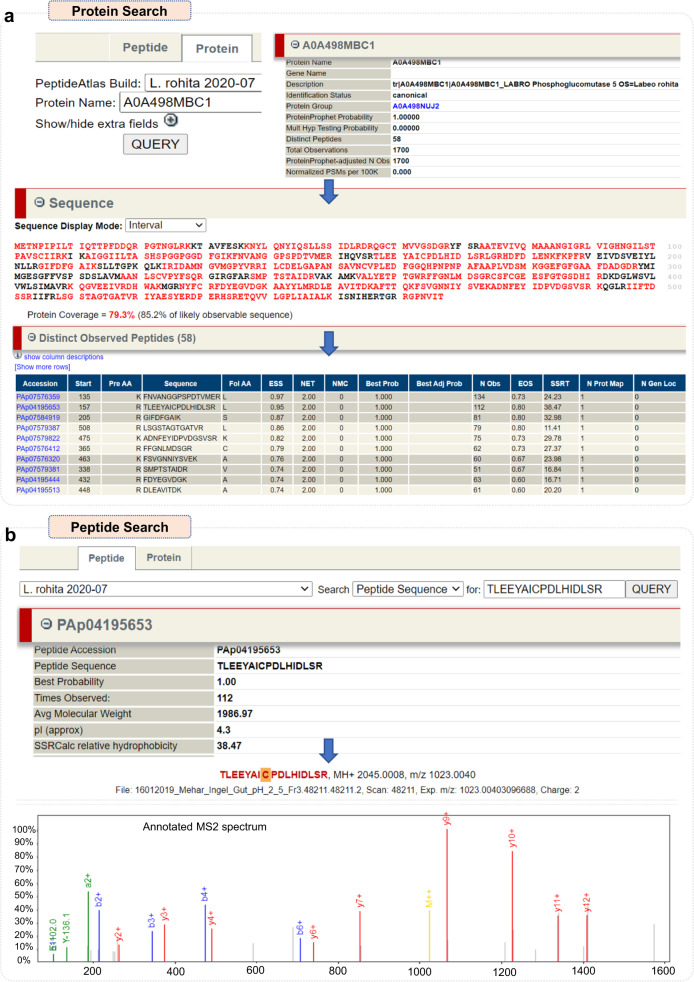
Example of a protein search and peptide search in Rohu PeptideAtlas. (**a**) Out of several collapsible sections for protein search, three are shown to provide an overview of protein information, observed peptides highlighted in red font and additional information for each observed peptide, respectively. (**b**) Under peptide view, two sections for one of the observed peptides of the same protein are shown representing general information about peptide and respective annotated MS2 spectrum where x-axis represents the m/z and y-axis shows the intensity.

For any targeted experiment, proteotypic peptides are the ideal targets which can be selected based on several scores assigned to a peptide in PeptideAtlas. For each protein entry, a dynamic page is obtained to provide mass spectral information and peptide modification details about the protein such as total observed peptides and a graphical representation of coverage of protein for each observed peptide. Additionally, all observed peptides are represented in a tabular format and ranked according to their empirical suitability score (ESS) empirical observability score (EOS) (Fig. 4a). ESS is a measure of incidence of observing a protein/peptide in a given sample while EOS represents how much suitable is the observed peptide for the significant proteotypic detection of protein from which it was obtained. Peptides having high value of EOS and map to a unique protein are the most suited candidates to monitor for identifying/quantifying a protein in a given sample. The protein view page also gives the information of all the tissues/sample in which the particular protein was detected.

For any observed peptide, a peptide view page presents all available information of respective peptide including its alignment to particular protein, genome mapping, modification site (if any). It also presents the peptide spectra in each sample where the peptide was observed (Fig. 4b). Spectral quality can be estimated based on the spectral information provided for each peptide in the Lorikeet spectral viewer. Peptide spectra along with the precursor mass and all product ion masses and detected product ions are presented in tabular format.

### Utility of PeptideAtlas information in SRM based targeted proteomic experiments

A set of peptides was taken for targeted verification using selected reaction monitoring (SRM) approach. Results were matched with the spectral library for the reliability of the data. This section shows the significance of PeptideAtlas in targeted experiments. We have performed targeted experiment for two proteins in female gonad tissue and similar kind of experiments can be designed and validated using PeptideAtlas information for all studied tissues of Rohu. Following steps were followed for SRM based verification experiment.

#### Generation of spectral library

PepXML (.pepXML) files obtained after comet search for female gonad sample were used to create a non-redundant spectral library. The spectral library was created using skyline software^34^ through the ‘build’ option under library tab inside the peptide settings. Finally, a .blib file was created and selected for the experiment.

#### Peptide and transition selection

Two proteins; Elongation factor 1 alpha (EF1 alpha-A0A498N236) and Zona pellucida sperm binding 3 like protein (zp3- A0A498NTM4) were selected for targeted verification. Only peptides unique to these proteins and without any missed cleavage were considered. Selected peptides were having ESS score greater than or equals to 0.4 and length ranged from 8 to 30 amino acids (Supplementary Table S3). Using skyline software, it was found that 593 transitions corresponding to 30 peptides and 44 precursors of the selected proteins were found in the spectral library of female gonad sample. Hence, two transition lists (TL1-305 transitions and TL2- 288 transitions) were exported for preparing the methods for performing SRM experiment (Supplementary Tables S4, S5).

#### Performing an SRM based targeted proteomics experiment

Instrument used for SRM experiment was Thermo Altis Triple quadrupole mass spectrometer. Transition lists for selected peptides were used to create respective targeted methods. Peptides obtained from female gonad tissue were run against the prepared methods in replicates (i.e., R1 and R2 for both the transition lists) with a liquid chromatography gradient of 10 minutes (See methods section). Data acquired was imported for further analysis in skyline against the same document from which the transition list was exported.

#### Validation of data/ spectral information using spectral library

A combination of multiple factors is generally used to correctly identify the peptides in a targeted experiment. The gold standard for this is heavy labelled peptides that co-elute with the peptide of interest. However, when heavy labelled peptides are unavailable as can be the case in most laboratory experiments, fragment ion matching to a spectrum library can be the best method to identify the peptide of interest unambiguously^39^. In case of spectral library matching, the observed spectra are matched with the existing spectra in the spectral library and a similarity score is calculated called as dot product (dotp). The dotp score is based on the normalised spectral contrast angle, which provides a measure of peak detection confidence. The dotp could range from 0 for lowest similarity to 1 for highest similarity and confident identification^40^.

**Fig. 5:**
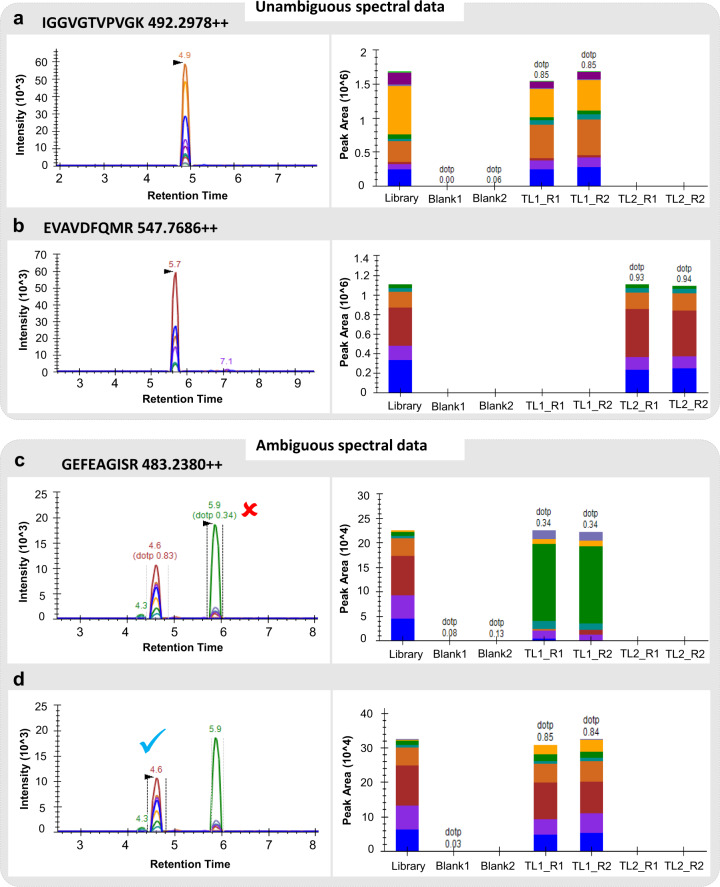
Targeted proteomic verification using spectral libraries. Left panel shows the peak view for the spectral information obtained for the peptide after performing SRM experiment and right panel shows the peak area view of the replicate runs along with match with the spectral library, (**a,b**) Spectral information for two peptides showing single, consistent peak with good match with library, (**c**) Wrongly annotated peak for the given peptide at 5.9 min with a dotp of 0.34 in both the replicate runs (right panel), (**d**) Correct annotated peak (4.6 min) based on the match with library (0.85/0.84) in both the replicates. [TL1 and TL2 represents the two transition lists, R1 and R2 represents the duplicate runs for the same sample].

In order to determine the promising peptides detected for the selected proteins; we imported the results to skyline. For the precursors, both singly and doubly charged product ions corresponding to y2 through last ion were considered. The spectral information was compared with the spectral library created from PeptideAtlas resource in order to confirm the reliability of the data. This was done based on the dot product metric (dotp) which is a measure of similarity between library spectra and query peaks^41^. Based on peak shape, peak area and co-elution of fragment ions, many peptides gave consistent results in both the replicate runs with a decent dotp value. Peak area and intensity values were consistent between the replicate runs and no peaks were observed in the blank runs. Table 4 shows respective dotp values for both doubly charged and/triply charged precursor of targeted peptides along with their ESS and EOS scores. For example, the peptide IGGVGTVPVGK and EVAVDFQMR were matched with the spectral library with a dotp value of more than 0.8 and 0.9, respectively in both the replicates (Fig. 5a,b). Similarly, a few more peptides exhibited single peaks for the respective peptide with no ambiguity.

**Table 4:** List of peptides selected for SRM based verification along with some details from PeptideAtlas and match score (dotp*) with spectral library.

Sequence	Accession	ESS	EOS	dotp ( + 2)	dotp ( + 3)
VFVDSCVATQAPDVNSLPR	PAp07598395	0.89	1.00	0.85	0.82
ALWSPMGMASALQSPFGVQEK	PAp07599055	0.78	0.33	0.85	0.77
QLLQGPVKPLDWR	PAp07598382	0.78	0.67	0.75	0.84
ADGAIVGVQCHYPR	PAp07598197	0.76	0.67	0.79	0.81
NMITGTSQADAALLIVSAAK	PAp04190051	0.75	0.26	0.76	0.76
YSFIENHGCFVDAK	PAp04184446	0.74	0.67	0.80	0.84
QPVTPSSVAVQCSEDR	PAp07601931	0.72	0.67	0.76	0.80
FPLVPEVQR	PAp07604998	0.69	0.83	0.88	NA
EVAVDFQMR	PAp07599266	0.68	0.50	0.93	NA
IETGVLKPGMVLTFSPAK	PAp04175036	0.62	0.32	0.79	0.85
SIEMHHQGLQTALPGHNVGFNIK	PAp04186345	0.60	0.26	NA	0.65
FMPQTQPEK	PAp07599143	0.59	0.50	0.82	NA
VGYSPVLDCHTTHVSCR	PAp04189673	0.55	0.37	0.56	0.70
ATFASVPSDAGR	PAp07604862	0.55	0.50	0.87	NA
TLLEVLDSLLPPVR	PAp07588809	0.54	0.42	0.80	0.85
IGGVGTVPVGK	PAp04171392	0.54	0.42	0.85	NA
LVPNKPLCVESFFHYPPLGR	PAp04189456	0.53	0.26	0.65	0.77
IHINLVIIGHVDSGK	PAp07601820	0.49	0.16	0.64	0.71
YTFTIIDAPGHR	PAp04188238	0.49	0.32	0.70	0.87
VYNHVPLR	PAp07604851	0.49	0.33	0.88	NA
MDLTEPPFSQK	PAp04189675	0.47	0.26	0.87	NA
STTTGHLVYK	PAp04190655	0.45	0.26	0.75	NA
GDVAGNAQQDPPSDVSSFIAQIIMLNHPGK	PAp04190359	0.44	0.16	NA	0.52
LEDWPQYLMSGDGATVK	PAp07599462	0.44	0.11	0.69	0.46
GEFEAGISR	PAp04185068	0.44	0.21	0.85	NA
IGFEIGAVPFIPVSGWSGENMIAPSQK	PAp07598582	0.43	0.11	NA	0.52
LMLDDWSYERPSNYYFLGNVFNLEASVK	PAp07599621	0.41	0.17	NA	0.47
VQFQLEAFMFQEGQSPSIYITCLLK	PAp07605170	0.40	0.17	NA	0.27
QLMVCVNK	PAp07602504	0.40	0.11	0.88	NA
GITIDISLLK	PAp04189685	0.39	0.16	0.82	NA

^*^dotp represents the measure of similarity between spectral library and experimental data.

However, there were several peptides for which multiple peaks scattered across the LC gradient were observed. These peaks were found to have good shape with co-elution, making it difficult to identify the correct peak in the absence of corresponding heavy labelled peptide. In such cases, spectral libraries play a significant role for determining the best match to obtain reliable and representative fragmentation patterns. For instance, for the peptide GEFEAGISR, two peaks were obtained in both the replicate runs, one at retention time 4.6 min and other at 5.9 min (Fig. 5c,d). Based on the match with spectral library (created using female gonad PepXML files) in both the runs, peak obtained at 4.6 would be the real peak as it has a dotp value of 0.85/0.84 compared to the one at 5.9 with a dotp of 0.34.

## Usage Notes

### Development and evaluation of a comprehensive PeptideAtlas for *Labeo rohita*

In the present study, we developed an open resource for fish proteome analysis for the scientific community based on high resolution mass spectrometry data from 19 different sample types of *L. rohita* (Rohu) using different protein extraction methods and sample fractionation. This is the first and foremost comprehensive fish proteome analysis (along with PTM information that is to be updated soon in PeptideAtlas as a part of another study). The complete building and evaluation process of the Rohu PeptideAtlas is explained elaborately in the Methods section.

### A valuable resource for designing targeted proteomics experiments

SRM or MRM based targeted proteomic experiments require unique transitions of the targeted proteins (or peptides) for accurate quantification. PeptideAtlas is the best resource for selecting the unique peptides and respective transitions using several tools in the PeptideAtlas. It also provides the information for best observable or identified tryptic peptides across wide range of sample types and also across different types of mass spectrometry. The interactive interface of PeptideAtlas helps to visualize individual and consensus spectra in PeptideAtlas to select and export either single or multiple targeted peptides/proteins and its respective transitions as.csv/.tsv format which can be imported directly into the mass spectrometry instrument for SRM/MRM experiment.

### A valuable resource for spectral library generation and data search

The Rohu PeptideAtlas built is dynamic and can be updated whenever a new proteomics dataset is generated in-house or get uploaded in the public repositories such as PRIDE, MASSIVE^42^ etc. The data repository in PeptideAtlas, TPP output files (.pepXML) used for generating PeptideAtlas and the results from PeptideAtlas can be used for generating spectral library using SpectraST, an integrated tool in TPP package. Spectral libraries are new generation peptide database with experimentally identified spectra used for the accurate and precise identification/quantification of peptides/proteins in DIA/SWATH analysis or for SRM/MRM data analysis.

### Best resource for Proteogenomic analysis and annotation

Accurate annotation of the genome is still a challenging task despite availability of advanced technology and algorithms. Integration of high-resolution mass spectrometry along with genomic data would improve the gene annotations. Rohu genome was sequenced recently and the preliminary annotations are available with no curation and it also contains several hypothetical proteins and pseudogenes^14^. Currently, in the UniProt database of *Labeo rohita*, only two proteins are reviewed which have protein evidence (PE) level 2 i.e., experimental evidence at transcript level. However, none of the protein has PE level 1 that represents the protein level evidence. The current dataset can help the UniProt curators, by providing the mass spectrometric based protein level evidence for the existence of *Labeo rohita* proteome. It has been reported that gene annotation can be improved with the help of mass spectrometric data^43,44^. Tanner *et al*. utilised the tandem mass spectra from human peptides and validated 11,000 introns and 39,000 exons at translation level along with identification of novel exons and splicing events^45^. In a similar manner, the peptide dataset provided in Rohu PeptideAtlas could help to improve the genome annotations and may provide evidence for pseudogenes, alternative splicing events, extended exons and hypothetical proteins.

## Supplementary information

Supplementary Figure 1

Supplementary File S1

Supplementary File S2

Supplementary File S3

Supplementary Table S1

Supplementary Table S2

Supplementary Table S3

Supplementary Table S4

Supplementary Table S5

## Data Availability

The authors do not have code specific to this work to disclose.
