# Supplementary material for: The PeptideAtlas of a widely cultivated fish Labeo rohita: A resource for the Aquaculture Community
Source: Sci Data. 2022 Apr 13;9:171. doi: 10.1038/s41597-022-01259-9 (PMC9008064; doi:10.1038/s41597-022-01259-9)
Supplement: Supplementary file 1 — Supplementary Figure 1 [file 41597_2022_1259_MOESM1_ESM.pdf]

SUPPLEMENTARY MATERIAL

Data Descriptor

The PeptideAtlas of a widely cultivated fish *Labeo rohita:* A resource for the Aquaculture Community

Mehar Un Nissa[1], Panga Jaipal Reddy[2$], Nevil Pinto[3$], Zhi Sun[2], Biplab Ghosh[4], Robert L. Moritz[2], Mukunda Goswami[3]* and Sanjeeva Srivastava[1]*

[1]Department of Biosciences and Bioengineering, Indian Institute of Technology Bombay, Powai, Mumbai 400076, India

[2]Institute for Systems Biology, Seattle, WA, 98109, USA

[3]Central Institute of Fisheries Education, Indian Council of Agricultural Research, Versova, Mumbai, Maharashtra 400061

[4]Regional Centre for Biotechnology, Faridabad, 121001, India

[$]Contributed equally

[*]Correspondence for fish work: Dr. Mukunda Goswami, E-mail: mukugoswami@gmail.com

[*]Correspondence for proteomics work: Dr. Sanjeeva Srivastava, E-mail: sanjeeva@iitb.ac.in, Phone: +91-22-2576-7779, Fax: +91-22-2572-3480

TABLE OF CONTENTS for SUPPLEMENTARY DATA

This manuscript contains **one** Supplementary figure, **three** supplementary files and **five** supplementary tables.

Supplementary Figures

Supplementary Figure	Caption
Figure S1	SDS-PAGE profile of all organs and peptide frequency

Supplementary Files

Supplementary files S1 to S3 are provided as separate documents.

Supplementary File	Caption	File format
File S1	Tissue wise details of all raw files	.pdf
File S2	The comet search parameter file	.pdf
File S3	MAYU statistical report	.xlsx

Supplementary Tables

Supplementary Table S1 to S5 are in .xlsx format and provided as separate excel file.

Supplementary Table	Caption	Number of worksheets
Table S1	List of all proteins in Rohu PeptideAtlas and EggNOG analysis details	4
Table S2	Experiment wise peptide list of Rohu PeptideAtlas and details of unique peptides and sequence coverage	2
Table S3	List of peptides and proteins taken for SRM verification experiment	1
Table S4	SRM Transition list1	1
Table S5	SRM Transition list2	1

Supplementary Figures

Supplementary Figure S1

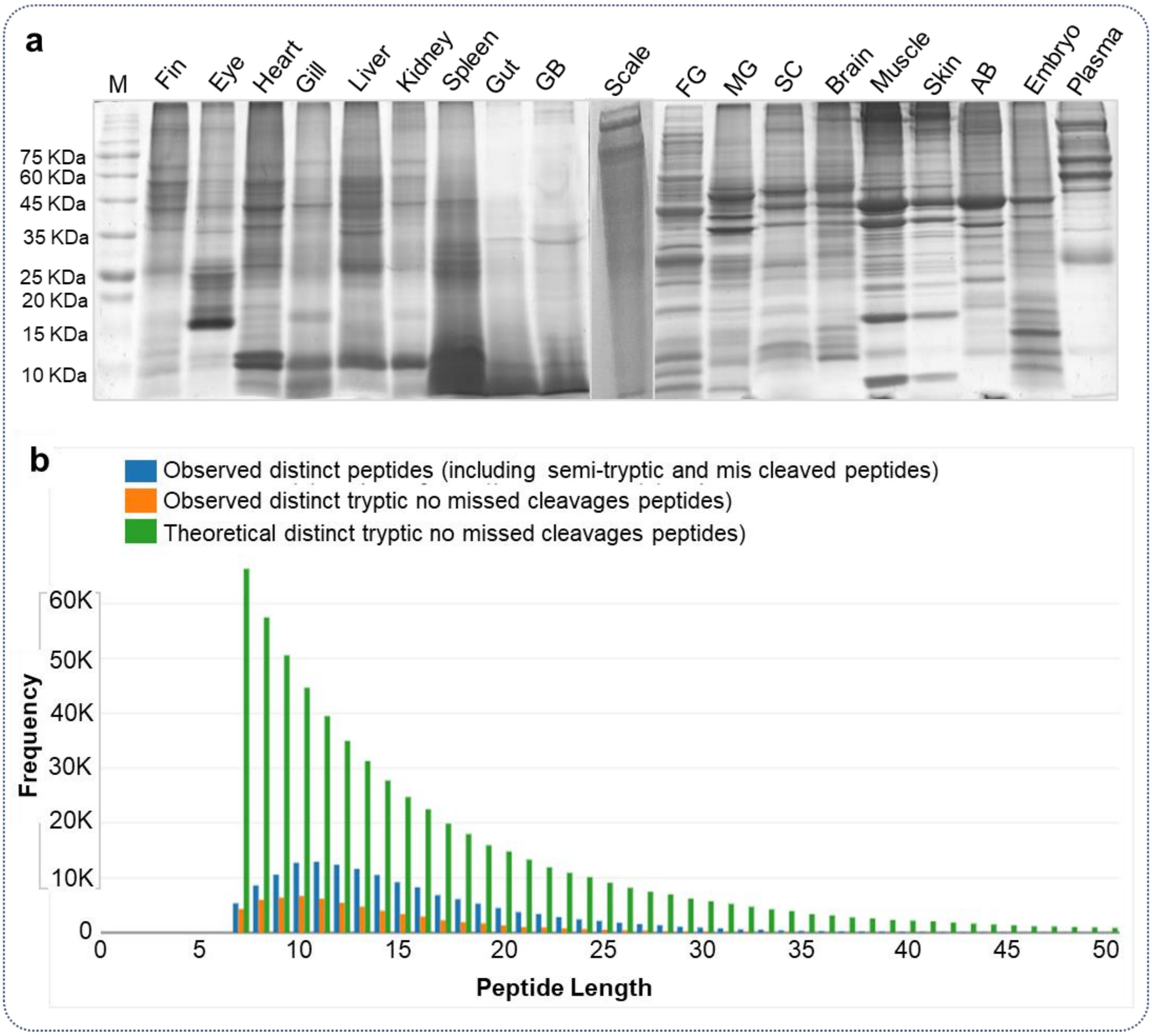

Supplementary Figure S1| SDS-PAGE profile of all organs and peptide frequency, **a.** Showing different band pattern across the organs (AB- Air bladder, GB- gall bladder, FG- female gonad, MG- male gonad, SC- spinal cord), **b.** Frequency of peptides with respect to missed cleavages

Supplementary Tables

Supplementary Table S1: List of all proteins in Rohu PeptideAtlas and EggNOG analysis details

This is an excel workbook and have four worksheets as below.

S.no.	Worksheet name	Description
1	Total_proteins_PA	Total 9286 proteins of peptide atlas (including 6015 canonical proteins)
2	Canonical proteins	Total canonical proteins
3	Canonical Protein eggNOG output	EggNOG output for ortholog analysis of all canonical proteins.
4	Input_Egg_NOG_Can	Input file for barplot of ortholog (related to Figure 2/Table 2)

Supplementary Table S2: Experiment wise peptide list of Rohu PeptideAtlas and details of unique peptides and sequence coverage.

This is an excel workbook and have two worksheets as below.

S.no.	Worksheet name	Description
1	Organ_wise_peptides	This sheet contains tissue/Experiment wise list of peptides enlisted in Rohu PeptideAtlas.
2	Distinct peptide_Coverage	This sheet contains the list of Canonical proteins with details like number of unique peptides (n_distinct peptides) and percent sequence coverage (Seq.coverage). Input for Figure 3e-f.

Supplementary Table S3: List of peptides and proteins taken for SRM verification experiment

This excel sheet contains the list of peptides along with some peptide wise information as obtained from Rohu PeptideAtlas

Supplementary Table S4: SRM Transition list1

This excel sheet is the transition list 1 that was used to create the method file for SRM run in Triple quadrupole mass spectrometer.

Supplementary Table S5: SRM Transition list2

This excel sheet is the transition list 1 that was used to create the method file for SRM run in Triple quadrupole mass spectrometer.
