# Supplementary material for: The PeptideAtlas of a widely cultivated fish Labeo rohita: A resource for the Aquaculture Community
Source: Sci Data. 2022 Apr 13;9:171. doi: 10.1038/s41597-022-01259-9 (PMC9008064; doi:10.1038/s41597-022-01259-9)
Supplement: Supplementary file 2 — Supplementary File S1 [file 41597_2022_1259_MOESM2_ESM.pdf]

Data Descriptor

The PeptideAtlas of a widely cultivated fish *Labeo rohita:* A resource for the Aquaculture Community

Mehar Un Nissa[1], Panga Jaipal Reddy[2$], Nevil Pinto[3$], Zhi Sun[2], Biplab Ghosh[4], Robert L. Moritz[2], Mukunda Goswami[3]* and Sanjeeva Srivastava[1]*

[1]Department of Biosciences and Bioengineering, Indian Institute of Technology Bombay, Powai, Mumbai 400076, India

[2]Institute for Systems Biology, Seattle, WA, 98109, USA

[3]Central Institute of Fisheries Education, Indian Council of Agricultural Research, Versova, Mumbai, Maharashtra 400061

[4]Regional Centre for Biotechnology, Faridabad, 121001, India

[$]Contributed equally

[*]Correspondence for fish work: Dr. Mukunda Goswami, E-mail: mukugoswami@gmail.com

[*]Correspondence for proteomics work: Dr. Sanjeeva Srivastava, E-mail: sanjeeva@iitb.ac.in, Phone: +91-22-2576-7779, Fax: +91-22-2572-3480

Supplementary File 1: Tissue wise details of all raw files

**S. no.**	**Raw file name**	**Tissue/ Experiment**
1	13042018_Fusion_Mehar_Airbladder_F1.raw	Air bladder
2	13042018_Fusion_Mehar_Airbladder_F2.raw	Air bladder
3	13042018_Fusion_Mehar_Airbladder_F3.raw	Air bladder
4	13042018_Fusion_Mehar_Airbladder_F4.raw	Air bladder
5	13042018_Fusion_Mehar_Airbladder_F5.raw	Air bladder
6	13042018_Fusion_Mehar_Airbladder_F6.raw	Air bladder
7	18012019_Mehar_Ingel_Air_bladder_pH_2_5_Fr1.raw	Air bladder
8	18012019_Mehar_Ingel_Air_bladder_pH_2_5_Fr2.raw	Air bladder
9	18012019_Mehar_Ingel_Air_bladder_pH_2_5_Fr3.raw	Air bladder
10	18012019_Mehar_Ingel_Air_bladder_pH_2_5_Fr4.raw	Air bladder
11	18012019_Mehar_Ingel_Air_bladder_pH_2_5_Fr5.raw	Air bladder
12	18012019_Mehar_Ingel_Air_bladder_pH_2_5_Fr6.raw	Air bladder
13	19012019_Mehar_Ingel_Air_bladder_pH_13_Fr1.raw	Air bladder
14	19012019_Mehar_Ingel_Air_bladder_pH_13_Fr2.raw	Air bladder
15	19012019_Mehar_Ingel_Air_bladder_pH_13_Fr3.raw	Air bladder
16	19012019_Mehar_Ingel_Air_bladder_pH_13_Fr4.raw	Air bladder
17	19012019_Mehar_Ingel_Air_bladder_pH_13_Fr5.raw	Air bladder
18	19012019_Mehar_Ingel_Air_bladder_pH_13_Fr6.raw	Air bladder
19	03112018_Mehar_Ingel_Brain_pH_2_5_Fr1.raw	Brain
20	03112018_Mehar_Ingel_Brain_pH_2_5_Fr2.raw	Brain
21	03112018_Mehar_Ingel_Brain_pH_2_5_Fr3.raw	Brain
22	03112018_Mehar_Ingel_Brain_pH_2_5_Fr4.raw	Brain
23	03112018_Mehar_Ingel_Brain_pH_2_5_Fr5.raw	Brain
24	03112018_Mehar_Ingel_Brain_pH_2_5_Fr6.raw	Brain
25	04112018_Mehar_Ingel_Brain_pH_13_Fr1.raw	Brain
26	04112018_Mehar_Ingel_Brain_pH_13_Fr2.raw	Brain
27	04112018_Mehar_Ingel_Brain_pH_13_Fr3.raw	Brain
28	04112018_Mehar_Ingel_Brain_pH_13_Fr4.raw	Brain
29	04112018_Mehar_Ingel_Brain_pH_13_Fr5.raw	Brain
30	04112018_Mehar_Ingel_Brain_pH_13_Fr6.raw	Brain
31	25012018_Fusion_Mehar_Ingel_Brain_F1.raw	Brain
32	25012018_Fusion_Mehar_Ingel_Brain_F2.raw	Brain
33	25012018_Fusion_Mehar_Ingel_Brain_F3.raw	Brain
34	25012018_Fusion_Mehar_Ingel_Brain_F4.raw	Brain
35	25012018_Fusion_Mehar_Ingel_Brain_F5.raw	Brain
36	25012018_Fusion_Mehar_Ingel_Brain_F6.raw	Brain
37	27052019_Fusion_2038_Mehar_Ingel_Embryo_Trizol_Fr1.raw	Embryo
38	27052019_Fusion_2038_Mehar_Ingel_Embryo_Trizol_Fr2.raw	Embryo
39	27052019_Fusion_2038_Mehar_Ingel_Embryo_Trizol_Fr3.raw	Embryo
40	27052019_Fusion_2038_Mehar_Ingel_Embryo_Trizol_Fr4.raw	Embryo
41	27052019_Fusion_2038_Mehar_Ingel_Embryo_Trizol_Fr5.raw	Embryo
42	29052019_Fusion_2038_Mehar_Ingel_Embryo_Trizol_Fr6.raw	Embryo
43	24112018_Mehar_Ingel_Eye_pH_13_Fr1.raw	Eye

44	24112018_Mehar_Ingel_Eye_pH_13_Fr2.raw	Eye
45	24112018_Mehar_Ingel_Eye_pH_13_Fr3.raw	Eye
46	24112018_Mehar_Ingel_Eye_pH_13_Fr4.raw	Eye
47	24112018_Mehar_Ingel_Eye_pH_13_Fr5.raw	Eye
48	24112018_Mehar_Ingel_Eye_pH_13_Fr6.raw	Eye
49	24112018_Mehar_Ingel_Eye_pH_2_5_Fr1.raw	Eye
50	24112018_Mehar_Ingel_Eye_pH_2_5_Fr2.raw	Eye
51	24112018_Mehar_Ingel_Eye_pH_2_5_Fr3.raw	Eye
52	24112018_Mehar_Ingel_Eye_pH_2_5_Fr4.raw	Eye
53	24112018_Mehar_Ingel_Eye_pH_2_5_Fr5.raw	Eye
54	24112018_Mehar_Ingel_Eye_pH_2_5_Fr6.raw	Eye
55	27012018_Fusion_Mehar_Ingel_Eye_F1.raw	Eye
56	27012018_Fusion_Mehar_Ingel_Eye_F2.raw	Eye
57	27012018_Fusion_Mehar_Ingel_Eye_F3.raw	Eye
58	27012018_Fusion_Mehar_Ingel_Eye_F4.raw	Eye
59	27012018_Fusion_Mehar_Ingel_Eye_F5.raw	Eye
60	27012018_Fusion_Mehar_Ingel_Eye_F6.raw	Eye
61	05042019_Mehar_2038_Ingel_Female_gonad_pH_8_Fr1.raw	Female gonad
62	05042019_Mehar_2038_Ingel_Female_gonad_pH_8_Fr2.raw	Female gonad
63	05042019_Mehar_2038_Ingel_Female_gonad_pH_8_Fr3.raw	Female gonad
64	05042019_Mehar_2038_Ingel_Female_gonad_pH_8_Fr4.raw	Female gonad
65	05042019_Mehar_2038_Ingel_Female_gonad_pH_8_Fr5.raw	Female gonad
66	05042019_Mehar_2038_Ingel_Female_gonad_pH_8_Fr6.raw	Female gonad
67	09042019_Mehar_2038_Ingel_Female_gonad_pH_2_5_Fr1.raw	Female gonad
68	09042019_Mehar_2038_Ingel_Female_gonad_pH_2_5_Fr2.raw	Female gonad
69	09042019_Mehar_2038_Ingel_Female_gonad_pH_2_5_Fr3.raw	Female gonad
70	09042019_Mehar_2038_Ingel_Female_gonad_pH_2_5_Fr4.raw	Female gonad
71	09042019_Mehar_2038_Ingel_Female_gonad_pH_2_5_Fr5.raw	Female gonad
72	09042019_Mehar_2038_Ingel_Female_gonad_pH_2_5_Fr6.raw	Female gonad
73	10042019_Mehar_2038_Ingel_Female_gonad_pH_13_Fr1.raw	Female gonad
74	10042019_Mehar_2038_Ingel_Female_gonad_pH_13_Fr3.raw	Female gonad
75	10042019_Mehar_2038_Ingel_Female_gonad_pH_13_Fr4.raw	Female gonad
76	10042019_Mehar_2038_Ingel_Female_gonad_pH_13_Fr5.raw	Female gonad
77	11042019_Mehar_2038_Ingel_Female_gonad_pH_13_Fr2.raw	Female gonad
78	11042019_Mehar_2038_Ingel_Female_gonad_pH_13_Fr6.raw	Female gonad
79	20092018_Mehar_Ingel_TRIzol_Female_Gonad_Fr1.raw	Female gonad
80	20092018_Mehar_Ingel_TRIzol_Female_Gonad_Fr2.raw	Female gonad
81	20092018_Mehar_Ingel_TRIzol_Female_Gonad_Fr3.raw	Female gonad
82	20092018_Mehar_Ingel_TRIzol_Female_Gonad_Fr4.raw	Female gonad
83	20092018_Mehar_Ingel_TRIzol_Female_Gonad_Fr5.raw	Female gonad
84	20092018_Mehar_Ingel_TRIzol_Female_Gonad_Fr6.raw	Female gonad
85	08042018_Fusion_Mehar_fish_Fin_F1.raw	Fin
86	08042018_Fusion_Mehar_fish_Fin_F2.raw	Fin
87	08042018_Fusion_Mehar_fish_Fin_F3.raw	Fin
88	08042018_Fusion_Mehar_fish_Fin_F4.raw	Fin
89	08042018_Fusion_Mehar_fish_Fin_F6.raw	Fin

90	08042018_Fusion_Mehar_fish_Fint_F5.raw	Fin
91	10042018_Fusion_Mehar_Gallbladder_F1.raw	Gall bladder
92	10042018_Fusion_Mehar_Gallbladder_F2.raw	Gall bladder
93	10042018_Fusion_Mehar_Gallbladder_F3.raw	Gall bladder
94	10042018_Fusion_Mehar_Gallbladder_F4.raw	Gall bladder
95	10042018_Fusion_Mehar_Gallbladder_F5.raw	Gall bladder
96	10042018_Fusion_Mehar_Gallbladder_F6.raw	Gall bladder
97	25112018_Mehar_Ingel_Gall_Bladder_Extract_TCA_Ac_Fr2.raw	Gall bladder
98	25112018_Mehar_Ingel_Gall_Bladder_Extract_TCA_Ac_Fr3.raw	Gall bladder
99	25112018_Mehar_Ingel_Gall_Bladder_Extract_TCA_Ac_Fr4.raw	Gall bladder
100	03022018_Fusion_Mehar_Gill_F1.raw	Gill
101	03022018_Fusion_Mehar_Gill_F2.raw	Gill
102	03022018_Fusion_Mehar_Gill_F3.raw	Gill
103	03022018_Fusion_Mehar_Gill_F4.raw	Gill
104	03022018_Fusion_Mehar_Gill_F5.raw	Gill
105	03022018_Fusion_Mehar_Gill_F6.raw	Gill
106	30012019_Mehar_Ingel_Gill_pH_2_5_Fr1.raw	Gill
107	30012019_Mehar_Ingel_Gill_pH_2_5_Fr2.raw	Gill
108	30012019_Mehar_Ingel_Gill_pH_2_5_Fr3.raw	Gill
109	30012019_Mehar_Ingel_Gill_pH_2_5_Fr4.raw	Gill
110	30012019_Mehar_Ingel_Gill_pH_2_5_Fr5.raw	Gill
111	30012019_Mehar_Ingel_Gill_pH_2_5_Fr6.raw	Gill
112	31012019_Mehar_Ingel_Gill_pH_13_Fr1.raw	Gill
113	31012019_Mehar_Ingel_Gill_pH_13_Fr2.raw	Gill
114	31012019_Mehar_Ingel_Gill_pH_13_Fr3.raw	Gill
115	31012019_Mehar_Ingel_Gill_pH_13_Fr4.raw	Gill
116	31012019_Mehar_Ingel_Gill_pH_13_Fr5.raw	Gill
117	31012019_Mehar_Ingel_Gill_pH_13_Fr6.raw	Gill
118	16012019_Mehar_Ingel_Gut_pH_2_5_Fr1.raw	Gut
119	16012019_Mehar_Ingel_Gut_pH_2_5_Fr2.raw	Gut
120	16012019_Mehar_Ingel_Gut_pH_2_5_Fr3.raw	Gut
121	16012019_Mehar_Ingel_Gut_pH_2_5_Fr4.raw	Gut
122	16012019_Mehar_Ingel_Gut_pH_2_5_Fr5.raw	Gut
123	16012019_Mehar_Ingel_Gut_pH_2_5_Fr6.raw	Gut
124	17012019_Mehar_Ingel_Gut_pH_13_Fr1.raw	Gut
125	17012019_Mehar_Ingel_Gut_pH_13_Fr2.raw	Gut
126	17012019_Mehar_Ingel_Gut_pH_13_Fr3.raw	Gut
127	17012019_Mehar_Ingel_Gut_pH_13_Fr4.raw	Gut
128	17012019_Mehar_Ingel_Gut_pH_13_Fr5.raw	Gut
129	17012019_Mehar_Ingel_Gut_pH_13_Fr6.raw	Gut
130	25012018_Fusion_Mehar_Ingel_Gut_F1.raw	Gut
131	25012018_Fusion_Mehar_Ingel_Gut_F2.raw	Gut
132	25012018_Fusion_Mehar_Ingel_Gut_F3.raw	Gut
133	25012018_Fusion_Mehar_Ingel_Gut_F4.raw	Gut
134	25012018_Fusion_Mehar_Ingel_Gut_F5.raw	Gut
135	25012018_Fusion_Mehar_Ingel_Gut_F6.raw	Gut

136	08112018_Mehar_Ingel_Heart_pH_2_5_Fr1.raw	Heart
137	08112018_Mehar_Ingel_Heart_pH_2_5_Fr2.raw	Heart
138	08112018_Mehar_Ingel_Heart_pH_2_5_Fr3.raw	Heart
139	08112018_Mehar_Ingel_Heart_pH_2_5_Fr4.raw	Heart
140	08112018_Mehar_Ingel_Heart_pH_2_5_Fr5.raw	Heart
141	08112018_Mehar_Ingel_Heart_pH_2_5_Fr6.raw	Heart
142	15112018_Mehar_Ingel_Heart_pH_13_Fr1.raw	Heart
143	15112018_Mehar_Ingel_Heart_pH_13_Fr2.raw	Heart
144	15112018_Mehar_Ingel_Heart_pH_13_Fr3.raw	Heart
145	15112018_Mehar_Ingel_Heart_pH_13_Fr4.raw	Heart
146	16112018_Mehar_Ingel_Heart_pH_13_Fr5.raw	Heart
147	16112018_Mehar_Ingel_Heart_pH_13_Fr6.raw	Heart
148	18032018_Fusion_Mehar_fish_Heart_F1.raw	Heart
149	18032018_Fusion_Mehar_fish_Heart_F2.raw	Heart
150	18032018_Fusion_Mehar_fish_Heart_F3.raw	Heart
151	18032018_Fusion_Mehar_fish_Heart_F4.raw	Heart
152	18032018_Fusion_Mehar_fish_Heart_F5.raw	Heart
153	18032018_Fusion_Mehar_fish_Heart_F6.raw	Heart
154	24102018_Mehar_Ingel_Kidney_pH_2_5_Fr1.raw	Kidney
155	24102018_Mehar_Ingel_Kidney_pH_2_5_Fr2.raw	Kidney
156	25102018_Mehar_Ingel_Kidney_pH_2_5_Fr3.raw	Kidney
157	25102018_Mehar_Ingel_Kidney_pH_2_5_Fr4.raw	Kidney
158	25102018_Mehar_Ingel_Kidney_pH_2_5_Fr5.raw	Kidney
159	25102018_Mehar_Ingel_Kidney_pH_2_5_Fr6.raw	Kidney
160	26102018_Mehar_Ingel_Kidney_pH_13_Fr1.raw	Kidney
161	27012018_Fusion_Mehar_Ingel_Kidney_F1.raw	Kidney
162	27012018_Fusion_Mehar_Ingel_Kidney_F2.raw	Kidney
163	27012018_Fusion_Mehar_Ingel_Kidney_F3.raw	Kidney
164	27012018_Fusion_Mehar_Ingel_Kidney_F4.raw	Kidney
165	27012018_Fusion_Mehar_Ingel_Kidney_F5.raw	Kidney
166	27012018_Fusion_Mehar_Ingel_Kidney_F6.raw	Kidney
167	27102018_Mehar_Ingel_Kidney_pH_13_Fr2.raw	Kidney
168	27102018_Mehar_Ingel_Kidney_pH_13_Fr3.raw	Kidney
169	27102018_Mehar_Ingel_Kidney_pH_13_Fr4.raw	Kidney
170	27102018_Mehar_Ingel_Kidney_pH_13_Fr5.raw	Kidney
171	27102018_Mehar_Ingel_Kidney_pH_13_Fr6.raw	Kidney
172	17032018_Fusion_Mehar_fish_liver_F1.raw	Liver
173	17032018_Fusion_Mehar_fish_liver_F2.raw	Liver
174	17032018_Fusion_Mehar_fish_liver_F3.raw	Liver
175	17032018_Fusion_Mehar_fish_liver_F4.raw	Liver
176	17032018_Fusion_Mehar_fish_liver_F5.raw	Liver
177	17032018_Fusion_Mehar_fish_liver_F6.raw	Liver
178	28102018_Mehar_Ingel_Liver_pH_2_5_Fr1.raw	Liver
179	28102018_Mehar_Ingel_Liver_pH_2_5_Fr2.raw	Liver
180	28102018_Mehar_Ingel_Liver_pH_2_5_Fr3.raw	Liver
181	28102018_Mehar_Ingel_Liver_pH_2_5_Fr4.raw	Liver

182	28102018_Mehar_Ingel_Liver_pH_2_5_Fr5.raw	Liver
183	28102018_Mehar_Ingel_Liver_pH_2_5_Fr6.raw	Liver
184	29102018_Mehar_Ingel_Liver_pH_13_Fr1.raw	Liver
185	29102018_Mehar_Ingel_Liver_pH_13_Fr2.raw	Liver
186	29102018_Mehar_Ingel_Liver_pH_13_Fr3.raw	Liver
187	29102018_Mehar_Ingel_Liver_pH_13_Fr4.raw	Liver
188	29102018_Mehar_Ingel_Liver_pH_13_Fr5.raw	Liver
189	29102018_Mehar_Ingel_Liver_pH_13_Fr6.raw	Liver
190	19082018_Mehar_Ingel_Male_Gonad_Fr1.raw	Male gonad
191	19082018_Mehar_Ingel_Male_Gonad_Fr2.raw	Male gonad
192	19082018_Mehar_Ingel_Male_Gonad_Fr3.raw	Male gonad
193	19082018_Mehar_Ingel_Male_Gonad_Fr4.raw	Male gonad
194	19082018_Mehar_Ingel_Male_Gonad_Fr5.raw	Male gonad
195	19082018_Mehar_Ingel_Male_Gonad_Fr6.raw	Male gonad
196	25112018_Mehar_Ingel_Male_gonad_pH_2_5_Fr1.raw	Male gonad
197	25112018_Mehar_Ingel_Male_gonad_pH_2_5_Fr2.raw	Male gonad
198	25112018_Mehar_Ingel_Male_gonad_pH_2_5_Fr3.raw	Male gonad
199	25112018_Mehar_Ingel_Male_gonad_pH_2_5_Fr4.raw	Male gonad
200	25112018_Mehar_Ingel_Male_gonad_pH_2_5_Fr5.raw	Male gonad
201	25112018_Mehar_Ingel_Male_gonad_pH_2_5_Fr6.raw	Male gonad
202	28112018_Mehar_Ingel_Male_gonad_pH_13_Fr1.raw	Male gonad
203	28112018_Mehar_Ingel_Male_gonad_pH_13_Fr2.raw	Male gonad
204	28112018_Mehar_Ingel_Male_gonad_pH_13_Fr3.raw	Male gonad
205	28112018_Mehar_Ingel_Male_gonad_pH_13_Fr4.raw	Male gonad
206	28112018_Mehar_Ingel_Male_gonad_pH_13_Fr5.raw	Male gonad
207	28112018_Mehar_Ingel_Male_gonad_pH_13_Fr6.raw	Male gonad
208	03022018_Fusion_Mehar_Muscle_F1.raw	Muscle
209	03022018_Fusion_Mehar_Muscle_F2.raw	Muscle
210	03022018_Fusion_Mehar_Muscle_F3.raw	Muscle
211	03022018_Fusion_Mehar_Muscle_F4.raw	Muscle
212	03022018_Fusion_Mehar_Muscle_F5.raw	Muscle
213	03022018_Fusion_Mehar_Muscle_F6.raw	Muscle
214	04082018_QE_Mehar_Muscle_Manu.raw	Muscle
215	09102018_Mehar_Ingel_Muscle_pH_2_5_Fr1.raw	Muscle
216	09102018_Mehar_Ingel_Muscle_pH_2_5_Fr2.raw	Muscle
217	09102018_Mehar_Ingel_Muscle_pH_2_5_Fr3.raw	Muscle
218	09102018_Mehar_Ingel_Muscle_pH_2_5_Fr4.raw	Muscle
219	09102018_Mehar_Ingel_Muscle_pH_2_5_Fr5.raw	Muscle
220	09102018_Mehar_Ingel_Muscle_pH_2_5_Fr6.raw	Muscle
221	10102018_Mehar_Ingel_Muscle_pH_13_Fr1.raw	Muscle
222	10102018_Mehar_Ingel_Muscle_pH_13_Fr2.raw	Muscle
223	10102018_Mehar_Ingel_Muscle_pH_13_Fr3.raw	Muscle
224	10102018_Mehar_Ingel_Muscle_pH_13_Fr4.raw	Muscle
225	10102018_Mehar_Ingel_Muscle_pH_13_Fr5.raw	Muscle
226	10102018_Mehar_Ingel_Muscle_pH_13_Fr6.raw	Muscle
227	20072018_QE_Mehar_Insol_Muscle_BGZT_1ug.raw	Muscle

228	20072018_QE_Mehar_Insol_Muscle_BGZT_600ng.raw	Muscle
229	18072019_Fusion_2038_Mehar_Fem_Plasma_Ingel_Fr1.raw	Female blood plasma
230	18072019_Fusion_2038_Mehar_Fem_Plasma_Ingel_Fr2.raw	Female blood plasma
231	18072019_Fusion_2038_Mehar_Fem_Plasma_Ingel_Fr3_4.raw	Female blood plasma
232	18072019_Fusion_2038_Mehar_Fem_Plasma_Ingel_Fr5_6.raw	Female blood plasma
233	18072019_Fusion_2038_Mehar_Fem_Plasma_Ingel_Fr7.raw	Female blood plasma
234	19072019_Fusion_2038_Mehar_Fem_Plasma_Ingel_Fr11.raw	Female blood plasma
235	19072019_Fusion_2038_Mehar_Fem_Plasma_Ingel_Fr8.raw	Female blood plasma
236	19072019_Fusion_2038_Mehar_Fem_Plasma_Ingel_Fr9_10.raw	Female blood plasma
237	07042018_Fusion_Mehar_fish_Scale_F1.raw	Scale
238	07042018_Fusion_Mehar_fish_Scale_F2.raw	Scale
239	07042018_Fusion_Mehar_fish_Scale_F3.raw	Scale
240	07042018_Fusion_Mehar_fish_Scale_F4.raw	Scale
241	07042018_Fusion_Mehar_fish_Scale_F5.raw	Scale
242	07042018_Fusion_Mehar_fish_Scale_F6.raw	Scale
243	08042018_Fusion_Mehar_fish_Scale_F1.raw	Scale
244	08042018_Fusion_Mehar_fish_Scale_F2.raw	Scale
245	08042018_Fusion_Mehar_fish_Scale_F3.raw	Scale
246	08112018_Mehar_Ingel_Scales_pH_2_5_Fr_1456_Pool.raw	Scale
247	08112018_Mehar_Ingel_Scales_pH_2_5_Fr2.raw	Scale
248	08112018_Mehar_Ingel_Scales_pH_2_5_Fr3.raw	Scale
249	16112018_Mehar_Ingel_Scales_pH_13_Fr2.raw	Scale
250	16112018_Mehar_Ingel_Scales_pH_13_Fr3.raw	Scale
251	16112018_Mehar_Ingel_Scales_pH_13_Fr_Pool_1456.raw	Scale
252	05012019_Mehar_Ingel_Skin_pH_13_Fr1.raw	Skin
253	05012019_Mehar_Ingel_Skin_pH_13_Fr2.raw	Skin
254	05012019_Mehar_Ingel_Skin_pH_2_5_Fr1.raw	Skin
255	05012019_Mehar_Ingel_Skin_pH_2_5_Fr2.raw	Skin
256	05012019_Mehar_Ingel_Skin_pH_2_5_Fr3.raw	Skin
257	07012019_Mehar_Ingel_Skin_pH_13_pool_Fr3456.raw	Skin
258	07012019_Mehar_Ingel_Skin_pH_2_5_pool_Fr456.raw	Skin
259	09042018_Fusion_Mehar_Skin_Fr1.raw	Skin
260	09042018_Fusion_Mehar_Skin_Fr2.raw	Skin
261	09042018_Fusion_Mehar_Skin_Fr3.raw	Skin
262	09042018_Fusion_Mehar_Skin_Fr4.raw	Skin
263	09042018_Fusion_Mehar_Skin_Fr5.raw	Skin
264	09042018_Fusion_Mehar_Skin_Fr6.raw	Skin
265	19032018_Fusion_Mehar_fish_Spinal_F1.raw	Spinal cord
266	19032018_Fusion_Mehar_fish_Spinal_F2.raw	Spinal cord
267	19032018_Fusion_Mehar_fish_Spinal_F3.raw	Spinal cord
268	19032018_Fusion_Mehar_fish_Spinal_F4.raw	Spinal cord
269	19032018_Fusion_Mehar_fish_Spinal_F5.raw	Spinal cord
270	19032018_Fusion_Mehar_fish_Spinal_F6.raw	Spinal cord
271	28122018_Mehar_Ingel_Spinal_cord_pH_2_5_Fr1.raw	Spinal cord
272	28122018_Mehar_Ingel_Spinal_cord_pH_2_5_Fr2.raw	Spinal cord
273	28122018_Mehar_Ingel_Spinal_cord_pH_2_5_Fr3.raw	Spinal cord

274	28122018_Mehar_Ingel_Spinal_cord_pH_2_5_Fr4.raw	Spinal cord
275	28122018_Mehar_Ingel_Spinal_cord_pH_2_5_Fr5.raw	Spinal cord
276	28122018_Mehar_Ingel_Spinal_cord_pH_2_5_Fr6.raw	Spinal cord
277	29122018_Mehar_Ingel_Spinal_cord_pH_13_Fr1.raw	Spinal cord
278	29122018_Mehar_Ingel_Spinal_cord_pH_13_Fr2.raw	Spinal cord
279	29122018_Mehar_Ingel_Spinal_cord_pH_13_Fr3.raw	Spinal cord
280	29122018_Mehar_Ingel_Spinal_cord_pH_13_Fr4.raw	Spinal cord
281	29122018_Mehar_Ingel_Spinal_cord_pH_13_Fr5.raw	Spinal cord
282	30122018_Mehar_Ingel_Spinal_cord_pH_13_Fr6.raw	Spinal cord
283	07082019_Fusion_2038_Mehar_Ingel_Spleen_pH_2_5_Fr1.raw	Spleen
284	07082019_Fusion_2038_Mehar_Ingel_Spleen_pH_2_5_Fr2.raw	Spleen
285	07082019_Fusion_2038_Mehar_Ingel_Spleen_pH_2_5_Fr3.raw	Spleen
286	07082019_Fusion_2038_Mehar_Ingel_Spleen_pH_2_5_Fr4.raw	Spleen
287	07082019_Fusion_2038_Mehar_Ingel_Spleen_pH_2_5_Fr5.raw	Spleen
288	07082019_Fusion_2038_Mehar_Ingel_Spleen_pH_2_5_Fr6.raw	Spleen
289	08082019_Fusion_2038_Mehar_Ingel_Spleen_pH_13_Fr1.raw	Spleen
290	09082019_Fusion_2038_Mehar_Ingel_Spleen_pH_13_Fr2.raw	Spleen
291	09082019_Fusion_2038_Mehar_Ingel_Spleen_pH_13_Fr3.raw	Spleen
292	09082019_Fusion_2038_Mehar_Ingel_Spleen_pH_13_Fr4.raw	Spleen
293	19032018_Fusion_Mehar_fish_Spleen_F1.raw	Spleen
294	19032018_Fusion_Mehar_fish_Spleen_F2.raw	Spleen
295	19032018_Fusion_Mehar_fish_Spleen_F3.raw	Spleen
296	19032018_Fusion_Mehar_fish_Spleen_F4.raw	Spleen
297	23032018_Fusion_Mehar_fish_Spleen_F5.raw	Spleen
298	23032018_Fusion_Mehar_fish_Spleen_F6.raw	Spleen
