# Supplementary material for: The PeptideAtlas of a widely cultivated fish Labeo rohita: A resource for the Aquaculture Community
Source: Sci Data. 2022 Apr 13;9:171. doi: 10.1038/s41597-022-01259-9 (PMC9008064; doi:10.1038/s41597-022-01259-9)
Supplement: Supplementary file 3 — Supplementary File S2 [file 41597_2022_1259_MOESM3_ESM.pdf]

Data Descriptor

The PeptideAtlas of a widely cultivated fish *Labeo rohita:* A resource for the Aquaculture Community

Mehar Un Nissa[1], Panga Jaipal Reddy[2$], Nevil Pinto[3$], Zhi Sun[2], Biplab Ghosh[4], Robert L. Moritz[2], Mukunda Goswami[3]* and Sanjeeva Srivastava[1]*

[1]Department of Biosciences and Bioengineering, Indian Institute of Technology Bombay, Powai, Mumbai 400076, India

[2]Institute for Systems Biology, Seattle, WA, 98109, USA

[3]Central Institute of Fisheries Education, Indian Council of Agricultural Research, Versova, Mumbai, Maharashtra 400061

[4]Regional Centre for Biotechnology, Faridabad, 121001, India

[$]Contributed equally

[*]Correspondence for fish work: Dr. Mukunda Goswami, E-mail: mukugoswami@gmail.com

[*]Correspondence for proteomics work: Dr. Sanjeeva Srivastava, E-mail: sanjeeva@iitb.ac.in, Phone: +91-22-2576-7779, Fax: +91-22-2572-3480

```
# comet_version 2017.01 rev. 0
# Comet MS/MS search engine parameters file.
# Everything following the '#' symbol is treated as a comment.
database_name =
/proteomics/jpanga/Mass_spec_data_analysis/Data_analysis/SS/Fish/fish_201
9/Labeo_DB_MK_DECOY_CONT.fasta
decoy_search = 0                       # 0=no (default), 1=concatenated
search, 2=separate search
peff_format = 0                        # 0=no (normal fasta, default),
1=PEFF PSI-MOD, 2=PEFF Unimod
peff_obo =                             # path to PSI Mod or Unimod OBO
file
num_threads = 8                      # 0=poll CPU to set num threads;
else specify num threads directly (max 64)
#
# masses
#
peptide_mass_tolerance = 20.00
peptide_mass_units = 2                 # 0=amu, 1=mmu, 2=ppm
mass_type_parent = 1                   # 0=average masses, 1=monoisotopic
masses
mass_type_fragment = 1                 # 0=average masses, 1=monoisotopic
masses
precursor_tolerance_type = 0           # 0=MH+ (default), 1=precursor
m/z; only valid for amu/mmu tolerances
isotope_error = 1                      # 0=off, 1=0/1 (C13 error),
2=0/1/2, 3=0/1/2/3, 4=-8/-4/0/4/8 (for +4/+8 labeling)
#
# search enzyme
#
search_enzyme_number = 1               # choose from list at end of this
params file
num_enzyme_termini = 1                 # 1 (semi-digested), 2 (fully
digested, default), 8 C-term unspecific , 9 N-term unspecific
allowed_missed_cleavage = 2            # maximum value is 5; for enzyme
search
#
# Up to 9 variable modifications are supported
# format:  <mass> <residues> <0=variable/else binary>
<max_mods_per_peptide> <term_distance> <n/c-term> <required>
#     e.g. 79.966331 STY 0 3 -1 0 0
#
variable_mod01 = 15.9949 MW 0 3 -1 0 0
variable_mod02 = 0.0 X 0 3 -1 0
variable_mod03 = 0.0 X 0 3 -1 0 0
variable_mod04 = 0.0 X 0 3 -1 0 0
variable_mod05 = 0.0 X 0 3 -1 0 0
variable_mod06 = 0.0 X 0 3 -1 0 0
variable_mod07 = 0.0 X 0 3 -1 0 0
variable_mod08 = 0.0 X 0 3 -1 0 0
variable_mod09 = 0.0 X 0 3 -1 0 0
max_variable_mods_in_peptide = 5
require_variable_mod = 0
#
# fragment ions
#
# ion trap ms/ms:  1.0005 tolerance, 0.4 offset (mono masses),
theoretical_fragment_ions = 1
```

```
# high res ms/ms:    0.02 tolerance, 0.0 offset (mono masses),
theoretical_fragment_ions = 0, spectrum_batch_size = 10000
#
fragment_bin_tol = 0.05               # binning to use on fragment ions
fragment_bin_offset = 0.0               # offset position to start the
binning (0.0 to 1.0)
theoretical_fragment_ions = 1           # 0=use flanking peaks, 1=M peak
only
use_A_ions = 0
use_B_ions = 1
use_C_ions = 0
use_X_ions = 0
use_Y_ions = 1
use_Z_ions = 0
use_NL_ions = 0                         # 0=no, 1=yes to consider NH3/H2O
neutral loss peaks
#
# output
#
output_sqtstream = 0                    # 0=no, 1=yes  write sqt to
standard output
output_sqtfile = 0                      # 0=no, 1=yes  write sqt file
output_txtfile = 0                      # 0=no, 1=yes  write tab-delimited
txt file
output_pepxmlfile = 1                   # 0=no, 1=yes  write pep.xml file
output_percolatorfile = 0               # 0=no, 1=yes  write Percolator
tab-delimited input file
print_expect_score = 1                  # 0=no, 1=yes to replace Sp with
expect in out & sqt
num_output_lines = 5                    # num peptide results to show
show_fragment_ions = 0                  # 0=no, 1=yes for out files only
sample_enzyme_number = 1                # Sample enzyme which is possibly
different than the one applied to the search.
                                        # Used to calculate NTT & NMC in
pepXML output (default=1 for trypsin).
#
# mzXML parameters
#
scan_range = 0 0                        # start and end scan range to
search; either entry can be set independently
precursor_charge = 0 0                  # precursor charge range to
analyze; does not override any existing charge; 0 as 1st entry ignores
parameter
override_charge = 0                     # 0=no, 1=override precursor
charge states, 2=ignore precursor charges outside precursor_charge range,
3=see online
ms_level = 2                            # MS level to analyze, valid are
levels 2 (default) or 3
activation_method = ALL                 # activation method; used if
activation method set; allowed ALL, CID, ECD, ETD, ETD+SA, PQD, HCD,
IRMPD
#
# misc parameters
#
digest_mass_range = 600.0 5000.0        # MH+ peptide mass range to
analyze
num_results = 100                       # number of search hits to store
internally
```

```
skip_researching = 1                    # for '.out' file output only,
0=search everything again (default), 1=don't search if .out exists
max_fragment_charge = 3                 # set maximum fragment charge
state to analyze (allowed max 5)
max_precursor_charge = 6                # set maximum precursor charge
state to analyze (allowed max 9)
nucleotide_reading_frame = 0            # 0=proteinDB, 1-6, 7=forward
three, 8=reverse three, 9=all six
clip_nterm_methionine = 0               # 0=leave sequences as-is; 1=also
consider sequence w/o N-term methionine
spectrum_batch_size = 10000                 # max. # of spectra to search
at a time; 0 to search the entire scan range in one loop
decoy_prefix = DECOY_                   # decoy entries are denoted by
this string which is pre-pended to each protein accession
output_suffix =                         # add a suffix to output base
names i.e. suffix "-C" generates base-C.pep.xml from base.mzXML input
mass_offsets =                          # one or more mass offsets to
search (values substracted from deconvoluted precursor mass)
#
# spectral processing
#
minimum_peaks = 10                      # required minimum number of peaks
in spectrum to search (default 10)
minimum_intensity = 0                   # minimum intensity value to read
in
remove_precursor_peak = 0               # 0=no, 1=yes, 2=all charge
reduced precursor peaks (for ETD), 3=phosphate neutral loss peaks
remove_precursor_tolerance = 1.5        # +- Da tolerance for precursor
removal
clear_mz_range = 0.0 0.0                # for iTRAQ/TMT type data; will
clear out all peaks in the specified m/z range
#
# additional modifications
#
add_Cterm_peptide = 0.0
add_Nterm_peptide = 0.0
add_Cterm_protein = 0.0
add_Nterm_protein = 0.0
add_G_glycine = 0.0000                  # added to G - avg.  57.0513,
mono.  57.02146
add_A_alanine = 0.0000                  # added to A - avg.  71.0779,
mono.  71.03711
add_S_serine = 0.0000                   # added to S - avg.  87.0773,
mono.  87.03203
add_P_proline = 0.0000                  # added to P - avg.  97.1152,
mono.  97.05276
add_V_valine = 0.0000                   # added to V - avg.  99.1311,
mono.  99.06841
add_T_threonine = 0.0000                # added to T - avg. 101.1038,
mono. 101.04768
add_C_cysteine = 57.021464              # added to C - avg. 103.1429,
mono. 103.00918
add_L_leucine = 0.0000                  # added to L - avg. 113.1576,
mono. 113.08406
add_I_isoleucine = 0.0000               # added to I - avg. 113.1576,
mono. 113.08406
add_N_asparagine = 0.0000               # added to N - avg. 114.1026,
mono. 114.04293
```

```
add_D_aspartic_acid = 0.0000           # added to D - avg. 115.0874,
mono. 115.02694
add_Q_glutamine = 0.0000               # added to Q - avg. 128.1292,
mono. 128.05858
add_K_lysine = 0.0000                  # added to K - avg. 128.1723,
mono. 128.09496
add_E_glutamic_acid = 0.0000           # added to E - avg. 129.1140,
mono. 129.04259
add_M_methionine = 0.0000              # added to M - avg. 131.1961,
mono. 131.04048
add_O_ornithine = 0.0000               # added to O - avg. 132.1610, mono
132.08988
add_H_histidine = 0.0000               # added to H - avg. 137.1393,
mono. 137.05891
add_F_phenylalanine = 0.0000           # added to F - avg. 147.1739,
mono. 147.06841
add_U_selenocysteine = 0.0000          # added to U - avg. 150.0379,
mono. 150.95363
add_R_arginine = 0.0000                # added to R - avg. 156.1857,
mono. 156.10111
add_Y_tyrosine = 0.0000                # added to Y - avg. 163.0633,
mono. 163.06333
add_W_tryptophan = 0.0000              # added to W - avg. 186.0793,
mono. 186.07931
add_B_user_amino_acid = 0.0000         # added to B - avg.   0.0000,
mono.   0.00000
add_J_user_amino_acid = 0.0000         # added to J - avg.   0.0000,
mono.   0.00000
add_X_user_amino_acid = 0.0000         # added to X - avg.   0.0000,
mono.   0.00000
add_Z_user_amino_acid = 0.0000         # added to Z - avg.   0.0000,
mono.   0.00000
#
# COMET_ENZYME_INFO _must_ be at the end of this parameters file
#
[COMET_ENZYME_INFO]
0.  No_enzyme              0      -           -
1.  Trypsin                1      KR          P
2.  Trypsin/P              1      KR          -
3.  Lys_C                  1      K           P
4.  Lys_N                  0      K           -
5.  Arg_C                  1      R           P
6.  Asp_N                  0      D           -
7.  CNBr                   1      M           -
8.  Glu_C                  1      DE          P
9.  PepsinA                1      FL          P
10. Chymotrypsin           1      FWYL        P
```
